# Walkable new urban LEED_Neighborhood-Development (LEED-ND) community design and children's physical activity: selection, environmental, or catalyst effects?

**DOI:** 10.1186/1479-5868-8-139

**Published:** 2011-12-20

**Authors:** Robert B Stevens,, Barbara B Brown

**Affiliations:** 1 Masters of Public Policy Program, University of Utah, Salt Lake City, UT 84112, USA; 2 Family and Consumer Studies Department, University of Utah, Salt Lake City, UT 84112, USA

**Keywords:** Walkability, new urbanism, LEED_ND, neighborhood design, walk to school, accelerometer

## Abstract

**Background:**

Interest is growing in physical activity-friendly community designs, but few tests exist of communities explicitly designed to be walkable. We test whether students living in a new urbanist community that is also a pilot LEED_ND (Leadership in Energy and Environmental Design-Neighborhood Development) community have greater accelerometer-measured moderate-to-vigorous physical activity (MVPA) across particular time periods compared to students from other communities. We test various time/place periods to see if the data best conform to one of three explanations for MVPA. Environmental effects suggest that MVPA occurs when individuals are exposed to activity-friendly settings; selection effects suggest that walkable community residents prefer MVPA, which leads to both their choice of a walkable community and their high levels of MVPA; catalyst effects occur when walking to school creates more MVPA, beyond the school commute, on schooldays but not weekends.

**Methods:**

Fifth graders (n = 187) were sampled from two schools representing three communities: (1) a walkable community, Daybreak, designed with new urbanist and LEED-ND pilot design standards; (2) a mixed community (where students lived in a less walkable community but attended the walkable school so that part of the route to school was walkable), and (3) a less walkable community. Selection threats were addressed through controlling for parental preferences for their child to walk to school as well as comparing in-school MVPA for the walkable and mixed groups.

**Results:**

Minutes of MVPA were tested with 3 × 2 (Community by Gender) analyses of covariance (ANCOVAs). Community walkability related to more MVPA during the half hour before and after school and, among boys only, more MVPA after school. Boys were more active than girls, except during the half hour after school. Students from the mixed and walkable communities--who attended the same school--had similar in-school MVPA levels, and community groups did not differ in weekend MVPA, providing little evidence of selection effects.

**Conclusions:**

Even after our controls for selection effects, we find evidence of environmental effects on MVPA. These results suggest that walkable community design, according to new urbanist and LEED_ND pilot design standards, is related to higher MVPA among students at certain times.

## Background

Insufficient physical activity (PA) relates to increased risk for child obesity and associated health problems [1]. In the U.S., objective accelerometer measures indicate that only 35% of girls and 49% of boys aged 6 to 11 attained the recommended minimum of moderate-to-vigorous PA (MVPA; 60 minutes × 5 days/week)[2]. Moreover, PA levels decline as children age [3,4]. PA levels of youth today are also lower than in years past, especially for active transportation modes to get to school [5]. Although 40.7% of U.S. children walked to school in 1969, only 12.9% walked in 2001 [6], a decline of over two-thirds. Special policy interventions might encourage children to walk to school [7], but community designs that encourage walking to school may have the benefit of working regardless of organized programs, thus potentially reaching more participants. This study tests whether a community designed with new urbanist and Leadership in Energy and Environmental Design-Neighborhood Development (LEED_ND) pilot features may reverse these trends of less physical activity among children.

### New urban and LEED_ND design goals

New urbanist designs vary across places but are intended to promote the behaviors and values articulated in the Charter for the New Urbanism, a document endorsed by the 4^th ^Congress on New Urbanism in 1996. These design goals generally include the "3Ds" of walkability: population Density, land use Diversity, and pedestrian friendly Designs [8]. When homes are on small lots near needed destinations like schools, the compact development form and proximity make walking trips feasible. When street designs avoid cul-de-sac forms in favour of more pedestrian-friendly, well-connected grid designs, trips become more direct and convenient. The Charter specifically promotes active transportation to school as a goal: "Schools should be sized and located to enable children to walk or bicycle to them" [9, p. 105]. Other design goals specified by the Charter include streets that are safe, comfortable, and interesting, and which encourage walking and neighborliness. Walkability goals are believed to be supported by a variety of design elements. These include narrowed streets with calmed traffic and sidewalks, as well as shallow front yards and front porches so that residents and passersby can see and speak with one another [10].

The U.S. Green Building Council originally launched Leadership in Energy and Environmental Design (LEED) certification programs to recognize buildings that provide green building features, such as energy and water efficiency. In 2007 it developed a pilot LEED_ND system, informed by the Charter for the New Urbanism, to recognize greener neighborhood plans and designs. The goal was to go beyond green buildings alone to certify the "greenness" of overall plans. Such plans reduce land consumption and automobile dependence as well as promote pedestrian activity, good air quality, and other livability and sustainability goals [11]. Relevant to the community in the present study, LEED-ND 2007 pilot criteria award points for pedestrian-friendliness features such as siting 50% of homes within 0.5 miles of school, lining streets with four-foot wide sidewalks, and bringing buildings close to the sidewalk [11,12]. Only general descriptions of the first LEED_ND communities are available [13]; this study will provide an initial look at physical activity among children in a community designed with new urban and LEED-ND pilot standards.

### Walkable design, walking to school, and physical activity

Adults within new urban communities have reported more neighborhood walking than those in standard suburban communities [14,15], but children living in communities designed by new urbanist standards have not yet been studied. However, studies do contrast children in walkable and non-walkable neighborhoods. In these studies, walkability is defined by one or more of the 3Ds of walkability, and the neighborhoods may be missing some elements of new urbanism. Compared to youth in neighborhoods with fewer of the 3Ds, youth in walkable neighborhoods in Atlanta, GA report more walking, especially white youth [16], and youth aged 12 to 15 [17]. Youth in Australia report more walking to school when their neighborhoods have well connected streets that are designed to handle relatively low traffic [18]. Finally, regardless of community design, proximity relates to more walking or active transportation [19-26]. However, walkability features aside from proximity to school have received only mixed support in two recent reviews [26,27]. Furthermore, California parents say their children play outdoors more when their children live on less walkable cul-de-sacs than on through streets [28]. Although well-known new urbanist Peter Calthorpe claims that kids "thrive" in less automobile dependent new urban communities [10], evidence is needed to support this claim. Walkable new urban and LEED_ND designs are increasing in popularity, but the behavioral and physical activity patterns in these community designs merit research attention [29].

We examine Daybreak, Utah, a newly developed new urbanist community designed by Fregonese-Calthorpe Associates [30], which is also a LEED_ND pilot community [11]. Accelerometer-measured levels of physical activity for the half hour before and after school and at other times defined below are gathered for fifth graders at Daybreak, called the "walkable community," and two control areas. "Mixed community" students reside in a suburb just outside Daybreak but attend school in Daybreak, making part of the path to school walkable; the term "mixed" reflects the fact that these students are exposed to standard suburban design at home, but they traverse the walkable community design en route to school, which might encourage active transportation to or from school. "Less walkable community" students live and attend school in an adjacent standard suburban community. Consistent with a review of past research, we also expect higher MVPA among boys compared to girls [31]. The ability to have walkable, mixed, and less walkable communities available for study enabled particular tests for three different explanations for physical activity patterns, as described below.

### Assessing selection, environmental, and catalyst effects

In research on child physical activity, three identified patterns of explanations for MVPA--selection, environmental, and catalyst--have been described and are summarized in Table 1. The selection model assumes that already active families may have been attracted to walkable Daybreak, but would likely have been active regardless of where they moved because of their preference for physical activity. This non-random selection into walkable neighborhoods, if not addressed in some way, may lead researchers to conclude that environmental effects exist when the true cause is a pre-existing preference for physical activity [32]. Thus Table 1 shows that, if active families select into walkable Daybreak, their children would be more active than other children across all settings that do not restrict physical activity. One standard way to handle this threat is to control for stated preferences [33,34]. Thus, we control for parental attitudes regarding whether they wish, ideally, their child would walk to school, as one way to adjust for selection.

**Table 1:** Patterns of MVPA for Walkable (W), Mixed, and Less Walkable (LW) groups across time periods: Illustrating hypothetical models and actual empirical support

	Supportive pattern for each time period			
Three models explaining MVPA	To/from school	In school	After school	Weekend
				
**1. Selection**: Pre-existing preferences for MVPA lead to both selection of the Walkable community & higher MVPA among Walkable community students across settings.	W > M, LW	W > M^a^	W > M, LW	W > M, LW
**2. Environment**: Walkable settings support higher MVPA among those exposed to walkable community settings.	W, M > LW	W = M^a^	W > M, LW	W > M, LW
**3. Catalyst**: MVPA associated with active transportation to school leads to more MVPA, both during the walk and later in the school day, but not on weekends, which lack the before school MVPA catalyst.	W > M > LW	W > M > LW	W > M > LW	W = M, LW

Actual results				
**Hybrid**: Results are most consistent with Environmental model (excluding weekends and girls after school)	W,^a ^M > LW	W = M = LW	W > M, LW, boys only	W = M, LW

^a ^School grounds were not assessed for physical activity opportunities so the comparison of W and M in these cells controls for any differences in school environments; however, results are the same if LW group is retained in the test.

^b ^p < .10; all other differences shown in actual results are p < .05

However, we also use strategic comparisons across the walkable, mixed, and less walkable community groups in this study to provide novel and more extensive tests of selection and competing models developed to explain MVPA. For example, we exploit the fact that walkable and mixed community students attend the same school. If walkable community students tend toward more MVPA due to selection preferences, these differences should emerge at school. In particular, lunch break is a school period when past research has demonstrated high between-child variability in MVPA, suggesting that lunch is a setting where MVPA preferences can be expressed [35,36]. We test whether Daybreak students show more MVPA during lunch and other school times (all other school times, such as class, recess, transitions between classes) than the mixed community children **at the same school**, which controls for environmental exposure to physical activity opportunities during school times (We did not audit physical activity supports on school grounds so leaving out the less walkable students in this comparison makes this test more precise due to the control over environmental exposure). If Daybreak children are more active during lunch or other school times, such as recess and transitions between classes, than their schoolmates from the mixed community, then some type of individual difference related to selection to community is a likely explanation.

The environmental model in row 2 of Table 1 assumes that exposure to walkable community design supports more MVPA. If true, then walkable Daybreak students should achieve more MVPA during school-day evenings and weekends, assuming that these are the times when they are exposed to their neighborhood environment. For the half hour before and after school, both the walkable and mixed community students may also experience more MVPA, given that both groups are exposed to the walkable community route, which might encourage active transportation to school. But during the school day, walkable Daybreak and mixed community students should not differ in MVPA because they are exposed to the same environment, a pattern shown in Table 1, row 2. Again, leaving out the Less Walkable students from this comparison increases precision, given that environmental exposure at school is held constant for the other two groups.

Finally, Cooper has identified a catalyst model that suggests that walks to school, regardless of cause, trigger or catalyze more activity throughout the school day, but not on weekends [37]. For example, compared to driven-to-school children, Danish walk-to-school children showed more MVPA during the walk to school and at other times during school days (school and free play for boys, after school for girls). But on weekends, both groups were similarly active [37]; without the catalyst of the walk to school the students who walk to school during the week achieve no MVPA advantage on weekends. A similar catalyst pattern emerged for boys, but not for girls, attending a British school [38]. Our groups are defined by neighborhood residence, not walking status, but we presume that groups with more self-reported walking (see Table 2) would be most likely to show a catalyst effect. Thus catalyst effects should be most apparent for walkable, then mixed, then less walkable community students, given the declining self-reported walk to school incidence across those groups (i.e., 87%, 58%, and 19% of students, respectively, say they walk to or from school at least once in a typical week).

**Table 2:** Covariates, child self-reported walks to or from school, and distances to school: Means (M) and standard deviations (SD)

	**Walkable community**		**Mixed community**		**Less walkable community**		**Boys**		**Girls**	
	**(n = 27)**		**(n = 75)**		**(n = 85)**		**(n = 79)**		**(n = 108)**	
	**M**	**SD**	**M**	**SD**	**M**	**SD**	**M**	**SD**	**M**	**SD**
	
Parent ideally wants child to walk (1-4)	3.89	0.58	3.07	1.14	2.57	1.20	3.05	1.14	2.90	1.22
Rooms in home (#)	7.93	2.77	8.08	2.32	9.12	2.35	8.32	2.60	8.68	2.34
Parent has some college (0,1)	0.81	0.40	0.83	0.38	0.74	0.44	0.78	0.41	0.79	0.41
Student reports typically walking to or from school at least once a week (0,1)	0.87	0.34	0.58	0.50	0.19	0.39	0.39	0.49	0.47	0.50
Single family detached home (0,1)	0.88	0.33	0.97	0.17	0.99	0.11	0.93	0.25	0.99	0.10
Distance to school (miles, GIS measures)	0.38	0.17	1.01	0.85	1.32	0.54	1.06	0.87	1.06	0.59

In sum, we test whether a) MVPA is higher for children in the walkable Daybreak community during out of school times when we assume they are exposed to the walkable community as well as for the mixed community children in the half hour before and after school (an environmental effect); b) MVPA is higher at school for Daybreak children than their mixed community counterparts at the same school and whether community differences remain significant when controlling for parental preferences (two ways to assess selection effects); c) MVPA is higher during school days, but not weekends, for groups that report more walking to/from school (a catalyst effect) and d) whether MVPA is higher for boys than for girls.

## Method

### Study procedures and sampling

The study was conducted in spring 2007 with fifth graders, who are typically 11 years old at the end of the U.S. academic year. Students from walkable, mixed, and less walkable communities in 11 classes at two schools participated with university and school IRB approval. After in-school study orientations, students took home then returned completed parental consents and parental surveys; children completed assent forms and surveys at school with researchers present to answer any questions. Parent surveys included parent preferences for their child to walk to school, sociodemographic variables, and addresses; child surveys included reports of walking to school. Students from walkable and mixed communities were distinguished by addresses reported by parents. Students wore ActiGraph GT1M accelerometers, a reliable brand [39]. Accelerometer data, collected in 30-second epochs, were gathered from 7:55 AM to 9:00 PM. Each minute was designated as MVPA when it was ≥ 3 metabolic equivalents (METs) according to the age-adjusted Freedson equation [40], which is validated for MVPA measures [41]. Students from both schools participated during the same weeks, which effectively controls for external factors such as weather.

Of the 335 names on the fifth grade attendance roster, 217 returned signed forms, with 6 parents opting out of participation and 211 agreeing to participate by the beginning of the study (62.99%). The 211 accelerometer data files were screened to assure valid hours (< 30 minutes of 0s)[42] and days (≥ 4 valid hours). Following Cooper et al., participants needed ≥ two valid week days for weekday analyses; they needed ≥ one valid day for weekend analyses[37]. These screens, including some accelerometer malfunctions, reduced the weekday sample size to 187. This represented a 55% and 57% response rate in the walkable and less walkable schools, respectively. Within the walkable school there were fewer participants living in the walkable than the mixed community (27 vs. 75), which reflected the fact that the walkable community had not yet completely developed. Many students did not wear the accelerometer on weekends, reducing the weekend sample size to 150; those without weekend wear were less active during the week days (82 vs. 103 MVPA average daily minutes) but were equally distributed across communities.

### Study sites and walkability

Although many homes were too new to be characterized by Census 2000 data, the subsequent Census 2010 data portray the area as a predominately home-owning, family-oriented area. The three neighborhoods in this study include residents from four census tracts. These four tracts, compared to the entire Salt Lake County population of > 1 million residents, have greater proportions of residents who are white (88.7% to 95% across the four tracts vs. 81.2% for the country) and lower proportions of residents who are Hispanic (5.1% to 8.2% vs. 17.1%). Consistent with the suburban location, most study area tracts have larger households (3.46 to 3.79 vs. 2.96 persons/household) and more owner-occupied housing (82.5% to 92.7% vs. 67.3%) than county-wide. As shown in Table 2, the parent surveys indicated that most homes were single-family detached (88%-99%).

Daybreak exhibits many new urbanist and LEED-ND walkability features. Detailed maps and visual images of the neighborhood, called Founders Park Village, are available on the Daybreak web site [43]. The homes were typically built with small lot sizes (median =.12 acres, obtained from parcel level data as Census 2000 data were too old to include this new community). Walkability features included no cul-de-sacs (a disconnected street form that reduces walking access). Daybreak also has road-separated walking paths and narrow streets with sidewalks leading to the centrally located school with adjacent community center. All participants in the community lived within one mile of the school (M = 0.39 miles, SD = 0.17). Mixed community children attended the walkable community school but lived just outside of the boundary of the walkable community, so their route to school had longer distances (M = 1.01 miles, SD = 0.85) and included a mixture of less walkable and walkable community designs. This community was a conventionally designed suburb with larger housing lot sizes (median = .25 acres), and many cul-de-sacs (12.76 per 100 developed acres of parcels). The participants within the less walkable community were also drawn from a conventionally designed suburban neighborhood/school area that was adjacent to the walkable school area. This community had the longest distances to school (M = 1.32 miles, SD= 0.54), largest lot sizes (median = .35 acres) and included multiple cul-de-sacs (6.56 per 100 acres). The school was sited in a less pedestrian friendly area--along a busy road with a sidewalk on only one side of the street.

In addition to these differences, an objective walkability audit using the Irvine Minnesota Inventory [44] and trained raters documented block-level walkability differences in the environments for all blocks between children's homes and the schools, as noted in prior published research [45]. In that study, Daybreak was more walkable in terms of block level indicators of traffic and crime safety, density and land-use diversity, and pleasantness. For example, all Daybreak homes had porches in front and sidewalks on both sides of the street; these requirements were not in effect in the other communities. Furthermore, a separate study confirmed that Daybreak children reported more walking (see Table 2) and that both Daybreak parents and children perceived their neighborhood to be more walkable [46]. This study tests whether objectively measured MVPA is more likely to be attained in a new urban community that past research has found to have more physical environmental walkability characteristics as well as parent and child perceived walkability.

### Control variables and statistical analyses

Parental preference was controlled by parents' answers (on a 4-point agreement scale) to the item, "Ideally my child would walk to school." Consistent with past research [47], parent education was controlled. Finally, as reported in a companion study of perceived barriers to walking [46], we controlled for fewer rooms per dwelling in the walkable community (see Table 2). Rooms per household may be a proxy for household income and was used because the school district did not allow questions about household income. Alternatively, rooms per household may reflect the more compact design standards of new urbanism.

Minutes of MVPA for targeted time intervals (i.e., 1/2 hour before school, school lunch, all other times at school, 1/2 hour after school, later on school nights, and weekends) were compared using 3 × 2 (Community by Gender) analyses of covariance (ANCOVAs), followed by simple effects tests of community differences. The most precise tests of selection and environmental effects are analyzed for the subsample of walkable and mixed community students when at the same school (a 2 × 2, Community by Gender design). These tests assure that students are exposed to the same environment, so that any differences in MVPA would reflect differences related to personal preferences for physical activity, which would support a selection effect.

## Results

### MVPA before and after school: environment and selection possibilities

Community design effects on MVPA are tested for 4 distinct out-of-school times when children may be exposed to their community design: 1/2 hour before school (when active transport to school is likely to occur), 1/2 hour after school, after school (from 1/2 hour after school until 9 PM), and weekends.

#### Before school

Before school, results showed significant main effects for community and gender, with no significant interaction (top half of Table 3). Boys achieved 1.54 more MVPA minutes than girls. According to simple effect contrasts, students in the walkable community achieved 1.86 more minutes of MVPA than students in the less walkable community, a marginally significant difference (*p *= .061). Students in the mixed community achieved 1.65 more MVPA minutes than students in the less walkable community, a significant difference.

**Table 3:** Main effects for MVPA minutes by Community and Gender: Analysis of covariance results and means (s.e.) adjusted for covariates

	**Walkable**	**Mixed**	**Less walkable**	**F**	**partial *η*^2^**	**Boys**	**Girls**	**F**	**partial *η*^2^**
	
Out of school									
1/2 hr. before school	6.93^b^	6.72^a^	5.07	3.76*	0.041	7.01	5.47	4.90**	0.027
	(0.86)	(0.46)	(0.44)			(0.44)	(0.54)		
1/2 hr. after school	9.13^a^	10.38^a^	6.35	21.07**	0.196	8.83	8.42	0.39	0.002
	(0.81)	(0.44)	(0.43)			(0.42)	(0.51)		
After school	45.31	44.13	46.03	0.13	0.002	51.64	37.68	14.61**	0.077
	(4.82)	(2.63)	(2.49)			(2.48)	(3.04)		
Weekend	31.72	25.83	29.66	0.89	0.013	34.33	23.82	8.12**	0.055
	(4.53)	(2.70)	(2.29)			(2.38)	(2.85)		
In school									
Lunch	14.06	13.76	14.32	0.20	0.002	16.98	11.11	37.74**	0.178
	(1.17)	(0.64)	(0.61)			(0.61)	(0.74)		
Other	32.26	32.72	33.35	0.07	0.001	35.98	29.57	7.73**	0.042
	(2.84)	(1.55)	(1.47)			(1.46)	(1.79)		

Note. Data collected in Utah, 2007. For main effects of community or gender, * *p *< .05; ** *p *< .01

Partial *η*^2 ^= partial eta squared

^a ^Cell is significantly (*p *< .05) different from less walkable.

^b ^Cell is marginally (*p *< .10) different from less walkable.

#### After school

In the 1/2 hour after school, results showed significant differences across communities, but not genders. Compared to the MVPA of students from the less walkable community, students from the walkable community accrued 2.78 more MVPA minutes (*p *< .01) and the students from the mixed community accrued 4.03 more MVPA minutes (*p *< .001).

The additional time for the students from the mixed community might reflect their greater distance from school, given that they are required to walk through part of the walkable community to get to school, as described earlier in the site descriptions. We did not control for distance to the school, given that proximity is intrinsic to walkable design; nevertheless, when the 1/2 hour before and after school tests are recomputed controlling distance to school, results are weakened (e.g., the F reduces from 21.07 to 18.76) but substantially the same, demonstrating that walkability effects involve more than proximity.

#### Afternoon/evening

After school (starting 1/2 hour after school), a significant main effect for gender was tempered by a significant gender by community interaction, *F*(2, 175) = 3.90, *p *= .02, *η*^2 ^= .043. Walkable community boys were especially active, with 61.68 adjusted MVPA minutes, compared to the 43.71 MVPA minutes average for all other combinations of gender and community, according to the significant follow-up tests (*p *= .028). Thus, walkable community boys engaged in about 18 more minutes of MVPA, compared to all other groups, in the after school period (starting 1/2 hour after school).

The additional time for the students from the mixed community might reflect their greater distance from school, given that they are required to walk through part of the walkable community to get to school, as described earlier in the site descriptions. We did not control for distance to the school, given that proximity is intrinsic to walkable design; nevertheless, when the 1/2 hour before and after school tests are recomputed controlling distance to school, results are substantially the same, demonstrating that walkability effects involve more than proximity.

#### Weekends

On weekends, the only significant result was that boys were more active than girls by 10.51 MVPA minutes per day.

### In-school MVPA: selection and environment

The most precise selection and environment model tests contrast MVPA for the walkable and mixed community groups because they control for school physical setting (bottom half of Table 3, first two columns). Walkable and mixed groups did not differ in MVPA during lunch or other school times, providing no support to a selection argument that walkable community children are predisposed to more MVPA when in settings where individual differences can be expressed (all *p *> 0.10). Consistent with the prior analysis of all three community groups, boys in the walkable and mixed community subgroups were more active than girls at lunch (see first two columns of Table 3, row 5; *F *(1,94) = 20.25, *p *< .001, *η*^2 ^= .177) and during other times of the school day (see first two columns of Table 3, row 6; *F *(1,94) = 5.32, *p *< .05, *η*^2 ^= .054), evidence that variability in PA levels within the same setting is possible. (Table 3 also demonstrates that the lack of difference in MVPA at school holds even if the third group of less walkable community students attending the other school is included in the analysis.)

As noted, one way to control for selection effects is to control parents' preferences that, ideally, their child would walk to school. Data were reanalyzed dropping that control variable from the analysis in order to understand its effect. The only difference that emerged was that the marginal effect becomes significant. That is, for the half hour before school, the model with the parental preference control shows walkable community children are marginally more active than less walkable community children (*p *= .061); this effect becomes significant (*p *=.007) when the parental preference control is dropped from the analysis. Therefore, if the parental preference represents pre-existing selection preferences, then some selection takes place, given that the effect of the walkable community residence is stronger when parental preference is absent from the model.

In sum, the results appear to support a "hybrid model," which is not a pure selection, environmental or catalyst model, as discussed below. The results of this hybrid model are summarized in the last row of Table 1.

## Discussion

The walkable community (Daybreak) children achieved 4.65 more minutes MVPA during the 1/2 hours before and after school than students from the less walkable community. The mixed community students achieved 5.66 more minutes of MVPA and may have benefitted from the fact that part of their route to school traversed the walkable community The longer walk perhaps accounted for their greater MVPA relative to Daybreak students. The walkable Daybreak community boys engaged in about 18 more MVPA minutes during the late afternoon/evening time period than children from all other groups. These results are consistent with claims that more walkable community designs support more physical activity.

Although selection threats are common in observational studies of community design, separate tests suggested that both selection and environmental influences operated in the study. Recall that selection in this study would occur if walkable Daybreak simply attracts more physically active residents. If pre-existing high activity levels and preferences cause these families to choose walkable Daybreak, family members should show greater MVPA across many settings, including at school, but this did not occur. If selection in the form of parental preferences for their children to walk to school causes the results, then controlling for these parental preferences should have reduced to insignificance all community differences, and it did not. It did reduce from significance (*p *=.007) to marginal significance (*p *= .061) the tendency for walkable Daybreak students to achieve more MVPA on the walk to school. Thus, like many other studies of selection effects in the walkable design and physical activity literature [32,48], selection may play some role, but does not account for all physical activity differences across communities. Also, it is possible parental preferences did not predate the move to walkable Daybreak; parents may have developed their preference for their child to walk to school after experiencing the walkable community. Ideally, attitudinal controls for selection would be measured prior to the move to the new environment, to see if the preferences really predated the move. Therefore, measuring parental preferences post-move may have controlled for reactions to environment rather than pre-existing preferences, making this an especially conservative statistical adjustment.

To provide more comprehensive tests of selection, future research is needed on large samples of households that are moving. Ideally, researchers could track pre- and post-move physical environmental features of walkability, physical activity supports such as parks, objective physical activity levels, and extensive attitudinal measures that assess why families move to new neighborhoods. A more extensive list of reasons for moving to a neighborhood could provide a more comprehensive assessment of attitudinal confounds. By tracking the moves of residents to more and less walkable neighborhoods, researchers could provide the strongest quasi-experimental study design, short of random assignment.

Of the models identified in Table 1, the results (row 4) show mixed support for all three, but strongest support for the environmental model. Consistent with the environmental effects model, walkable Daybreak and mixed community children are more active during the 1/2 hours before and after school, when likely exposed to the walkable features of Daybreak (effects are marginally significant in favor of Daybreak students in the 1/2 hour before school, but significant for the 1/2 hour after school). Walkable Daybreak boys--but not girls--are more active during school-day late afternoons/evenings. No weekend differences emerged across communities; perhaps the mixed and less walkable community students go to more PA-friendly areas on weekends; GPS tracking could address this possibility.

Boys were often more active than girls, a common finding [49,50]. However, the gender gap was closed for the half hour after school. Given pervasive gender differences in PA, any settings that overcome those differences are of particular value. The greater MVPA on school evenings for walkable Daybreak boys but not girls suggests a gender by environment interaction, consistent with some past research. For example, in past research, PA was higher for boys living near park and recreational land [51], but higher for girls living near private recreational facilities [52]. New urban designers and health advocates need more research to understand gender-specific appeals of PA design supports in their communities.

Notice that this study contrasted MVPA differences across communities, rather than the more typical test of MVPA differences between walkers and non-walkers to school. It should be more difficult to detect MVPA differences across communities compared to detecting differences across groups defined by whether they engage in the physical activity of walking to school or not. Yet the extra 4.65 minutes during the 1/2 hour before/after school for walkable Daybreak students, computed for the whole group, is within the range of differences other studies have found when they compare walkers to non-walkers. Walkers' extra minutes in past research include: 3.5 in the to-school hour in Denmark [37]; 8-14 in the 2 hours before/after school in England [38]; 5.98 and 9.77 (for 0.5-1 mile and 1-5 mile distances from home to school, respectively) in England [53]; 3.2 for to-school, 4.7 for from-school, and 9.5 for to/from school walkers in six U.S. states [54]; and about 10 minutes for walking to/from school in South Carolina among regular walkers [55].

Longitudinal studies are needed to address the inherent weakness of our cross-sectional design, although this study provided more comprehensive tests of the selection threat than do many cross-sectional studies. Sample sizes are also small, especially at walkable Daybreak, which limits power. The school district also restricted survey length, which limited controls for other potential covariates, such as income. GPS data were also not available to confirm where PA occurred. Finally, we consider the possibility that parental preference controls yield conservative results, given that those preferences may have arisen after the move to the community, instead of indexing pre-existing preferences.

## Conclusions

This research adds to the findings that community design can relate to child physical activity and does so for an increasingly popular new community design--new urbanism. Although the Centers for Disease Control and Prevention's (CDC's) Task Force on Community Preventive Services [56] has already recommended walkable community designs, their one study of children in support of that recommendation involved observations of German children using a street more after pedestrian friendly traffic calming measures were introduced [57]. This study bolsters the CDC recommendation with objectively measured physical activity data from U.S. children. This additional evidence is especially important in light of current pressures toward "sprawl schools" [58]. U.S. school districts often require large tracts of land to build schools and these tracts are often only affordable and available on the distant fringe of urban development. If these results can be replicated, then school districts may have sufficient evidence to endorse new urbanist or LEED_ND design standards for schools, which involve more central locations with more walkable routes to nearby homes. By 2030, 50% of all U.S. buildings are projected to have been built or renovated since 2000 [59]; this will create many opportunities for developing new and retrofitted walkable communities.

## List of abbreviations

ANCOVA: analysis of covariance; CDC: Centers for Disease Control and Prevention; LEED_ND: Leadership in Energy and Environmental Design-Neighborhood Development; MVPA Moderate-to-vigorous physical activity.

## Competing interests

The authors declare that they have no competing interests.

## Authors' contributions

BB conceptualized the study and RS developed coding for the accelerometer data. Both BB and RS participated in writing, data analysis, and editing. Both authors read and approved the final manuscript.
